# Identification of serum glycoprotein ligands for the immunomodulatory receptor blood dendritic cell antigen 2

**DOI:** 10.1093/glycob/cwy050

**Published:** 2018-06-11

**Authors:** Jong-won Kim, James Budzak, Yu Liu, Sabine A F Jégouzo, Kurt Drickamer, Maureen E Taylor

**Affiliations:** Department of Life Sciences, Sir Ernst Chain Building, Imperial College, London, UK

**Keywords:** C-type lectin, glycan-binding protein, immunoglobulin, proteomics, serum glycoproteins

## Abstract

Blood dendritic cell antigen 2 (BDCA-2) is a C-type lectin found on the surface of plasmacytoid dendritic cells. It functions as a glycan-binding receptor that downregulates the production of type I interferons and thus plays a role in oligosaccharide-mediated immunomodulation. The carbohydrate recognition domain in BDCA-2 binds selectively to galactose-terminated bi-antennary glycans. Because the plasmacytoid dendritic cells function in a plasma environment rich in glycoproteins, experiments have been undertaken to identify endogenous ligands for blood dendritic cell antigen 2. A combination of blotting, affinity chromatography and proteomic analysis reveals that serum glycoprotein ligands for BDCA-2 include IgG, IgA and IgM. Compared to binding of IgG, which was previously described, IgA and IgM bind with higher affinity. The association constants for the different subclasses of immunoglobulins are below and roughly proportional to the serum concentrations of these glycoprotein ligands. Binding to the other main serum glycoprotein ligand, α_2_-macroglobulin, is independent of whether this protease inhibitor is activated. Binding to all of these glycoprotein ligands is mediated predominantly by bi-antennary glycans in which each branch bears a terminal galactose residue. The different affinities of the glycoprotein ligands reflect the different numbers of these galactose-terminated glycans and their degree of exposure on the native glycoproteins. The results suggest that normal serum levels of immunoglobulins could downmodulate interferon stimulation of further antibody production.

## Introduction

Blood dendritic cell antigen 2 (BDCA-2) is a specific marker for plasmacytoid dendritic cells circulating in the blood (Dzionek et al. 2000, 2001). These cells play a key role in response to both viral and bacterial antigens by providing a source of type I interferon (IFN), which stimulates direct antiviral functions and can also enhance the activity of dendritic cells and T and B lymphocytes (Reizis et al. 2011; Schraml and Reis e Sousa 2015; Swiecki and Colonna 2015). The effects of type I IFN are complex and can be detrimental as well as protective, so its production must be regulated. Crosslinking of BDCA-2 leads to inhibition of the IFN response by raising cytoplasmic Ca^2+^ through a pathway involving tyrosine-protein kinase BTK and phospholipase γ2 (Cao et al. 2007; Röck et al. 2007; Jähn et al. 2010). The BDCA-2 polypeptide consists of an extracellular C-type carbohydrate recognition domain (CRD) linked to a transmembrane domain and a short cytoplasmic sequence that lacks signaling motifs (Dzionek et al. 2001). The BDCA-2 polypeptide is associated with the common Fc receptor γ subunit (FcRγ). An immunotyrosine activation motif in the cytoplasmic domain of FcRγ interacts with Syk kinase to initiate the Ca^2+^ mobilization pathway (Cao et al. 2007; Röck et al. 2007; Plato et al. 2013).

The ability of a monoclonal antibody to BDCA-2 to inhibit type I IFN secretion has been exploited as a potential therapy for the autoimmune disease systemic lupus erythematosus (Pellerin et al. 2015). However, studies of BDCA-2 signaling have relied on the use of the monoclonal antibody to initiate signaling, since natural ligands for the receptor are not known. Structural and glycan array analyses of the CRD from BDCA-2 reveal high specificity for the sequence Galβ1-4GlcNAcα1-2Man at the nonreducing end of oligosaccharides (Jégouzo et al. 2015). Natural glycans bearing this sequence are typically N-linked oligosaccharides. In many such glycans, the galactose residues are sialylated, preventing binding to BDCA-2. However, there are glycoproteins, such as serum IgG, that contain a significant portion of the unsialylated, galactose-terminated glycans (Parekh et al. 1988; Yamada et al. 1997; Shikata et al. 1998; Wuhrer et al. 2007). IgG has been shown to bind to BDCA-2 (Jégouzo et al. 2015).

The fact that IgG binds to BDCA-2 suggests that other serum glycoproteins might also be potential ligands. In the work reported here, a combination of blotting, affinity chromatography and proteomic experiments has been used to identify additional serum glycoprotein ligands for BDCA-2, including IgA, IgM and α_2_-macroglobulin. The serum concentrations of these glycoproteins exceed their affinity for BDCA-2.

## Results

### Identification of glycoprotein ligands for BDCA-2

**Fig. 1.**
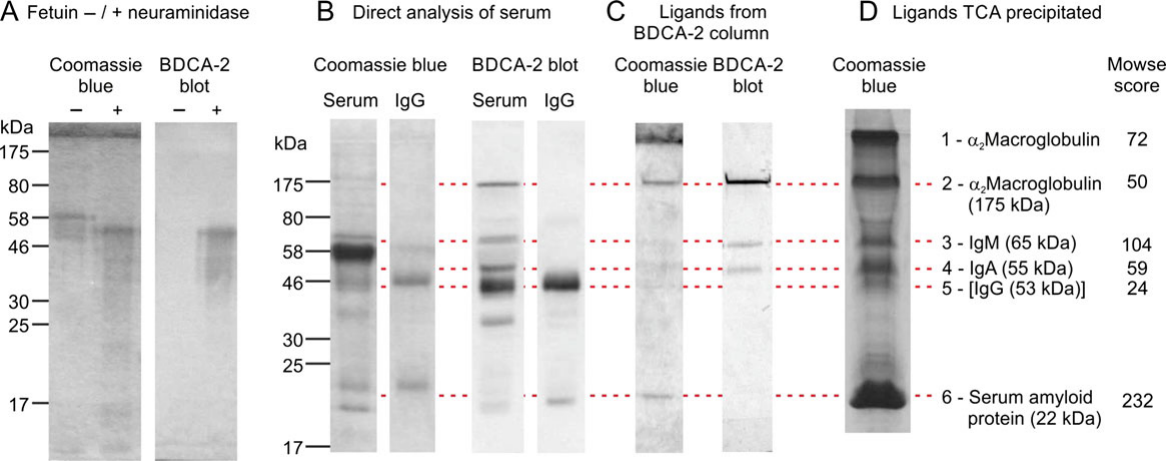
Identification of serum glycoprotein ligands for BDCA-2. (**A**) Demonstration of blotting specificity with samples of fetuin and asialofetuin. Aliquots (2 μg) were resolved on a 17.5% SDS polyacrylamide gel and either stained with Coomassie blue or blotted onto nitrocellulose and probed with CRD from BDCA-2 complexed with streptavidin–alkaline phosphatase. (**B**) Direct probing for glycoprotein ligands in serum. Aliquots corresponding to 0.05 μL of serum and 0.5 μg of human IgG were resolved on a 12.5% SDS polyacrylamide gel. Separate lanes were stained with Coomassie blue or blotted onto nitrocellulose and probed with CRD from BDCA-2 complexed with streptavidin–alkaline phosphatase. (**C**) Analysis of glycoprotein ligands following affinity chromatography on immobilized CRD. Aliquots of proteins eluted from the affinity column with EDTA were run in parallel and stained and blotted as in (**A**). (**D**) Separation of affinity-purified glycoprotein ligands following precipitation with trichloroacetic acid. Gel was stained with Coomassie blue and bands 1–6 were excised for proteomic analysis. Band 1 corresponds to material that did not enter the separating gel following precipitation. Proteins identified in each band are indicated along with their molecular weights (Table I). EDTA, ethylenediaminetetraacetic acid.

Glycan array analysis, binding competition studies and structural studies of the binding specificity of BDCA-2 indicated that the CRD binds with highest affinity to galactose-terminated N-linked glycans (Jégouzo et al. 2015). Because IgG is known to bear a significant fraction of such glycans, it was suggested that IgG is a potential ligand for BDCA-2 in serum, and binding to IgG was demonstrated. To obtain a broader picture of potential glycoprotein ligands in serum, blotting and affinity chromatography experiments have been undertaken. Blotting experiments employed biotin-tagged CRD from BDCA-2, in which a 12-amino acid biotinylation sequence has been appended to the C-terminus. Following co-expression with biotin ligase in bacterial cells, biotin is appended to a single lysine residue, which allows the formation of a tetravalent complex with streptavidin conjugated to alkaline phosphatase (Jégouzo et al. 2015). To demonstrate the specificity of the blotting, control samples of fetuin and neuraminidase-treated fetuin were separated on SDS polyacrylamide gels, transferred to nitrocellulose membranes and incubated with BDCA-2 complexed with alkaline phosphatase-conjugated streptavidin (Figure 1A). Only the neuraminidase-digested sample binds to the BDCA-2 complex, as expected from the specificity of BDCA-2 for galactose-terminated glycans.

**Table I.** Proteomic identification of serum proteins bound to immobilized BDCA-2

Band	Rank				
	1	2	3	4	5
1	**A2M (72)**	**IGH (18)**	AKAP17A (18)	TCFL5 (18)	TMEM74 (18)
2	**A2M (50)**	BCKDHA (21)	ARMC8 (17)	CP (16)	ST8SIA4 (16)
3	**IGHM (104)**	ZC3H18 (33)	SYNE2 (23)	ADCK5 (23)	ZNF16 (21)
4	**IGHA1 (59)**	**IGHA2 (59)**	RAD51 (22)	KLHL34 (19)	ABCA3 (19)
5	DMKN (24)	**IGH1/2/3 (21)**	ZNF274 (19)	CLK2 (17)	ZMYM2 (17)
6	**APCS (232)**	MT-ATP6 (33)	ACSBG1 (30)	ZNF622 (20)	KIF2A (18)

Proteins are identified by gene numbers in the National Center for Biotechnology Information database, with Mowse scores indicated in parentheses. Scores over 42 are significant at *P* < 0.05. Band numbers refer to Figure 1. In band 5, DMKN is a likely contaminant from skin. Serum proteins are highlighted in bold. DMKN, dermokine .

Total human serum proteins were resolved by SDS polyacrylamide gel electrophoresis, blotted and probed with the BDCA-2 complex (Figure 1B). Compared to the results for pure IgG, which was run in parallel for comparison, the results indicate that IgG is a prominent serum glycoprotein ligand for BDCA-2. There are strong signals for additional bands, but, major serum components such as serum albumin do not bind BDCA-2, indicating that the observed bands on the blot represent glycan-specific binding.

Affinity chromatography on immobilized CRD from BDCA-2 was used to isolate glycoprotein ligands from serum. Glycoproteins bound in the presence of Ca^2+^ and eluted with EDTA were resolved by SDS polyacrylamide gel electrophoresis (Figure 1C). Bands detected with BDCA-2 correspond to the bands detected in serum, with the exception that the intensity of the IgG band is reduced. Bands stained with Coomassie blue largely correspond to the bands detected on the blots, although the relative intensities are different.

The bands were excised, reduced and carboxymethylated, and digested with trypsin for analysis by mass spectrometry (MS). Proteomic analysis against the human protein database provided clear identification of all of the major bands (Figure 1D and Table I). The results indicate the presence of the heavy chains of IgA and IgM, α_2_-macroglobulin and serum amyloid protein. The peptides matched for IgA are common to both IgA1 and IgA2 subtypes. The band corresponding to IgG on the blots was too weak for definitive identification by MS, but it was detected as the second best match in this band. In each case, the apparent molecular weights of the main bands correspond to the intact molecular weights of these polypeptides. Following the acid precipitation, some of the α_2_-macroglobulin fails to enter the gel and several lower molecular weight bands appear to be proteolytic fragments of these proteins, particularly IgA and α_2_-macroglobulin.

Differences in the intensities of the signals between the blots and the isolated proteins reflect the fact that the signals in each case represent the effects both of abundance of the proteins in serum and their relative affinities for BDCA-2. The affinities in turn are affected by the way in which glycans are presented to the receptor. In some cases, glycans displayed on denatured glycoproteins on nitrocellulose are likely to be more accessible than when they are presented on folded proteins in the case of the affinity chromatography. The results indicate that IgG is not the only glycoprotein ligand in serum, but additional analysis is required to assess the extent to which different serum proteins are likely to interact with BDCA-2 under native conditions.

Although serum amyloid protein is significantly enriched on the affinity column, it appears only as a very weak band on the serum blot and is almost undetectable in the blot of affinity-purified material. The fact that serum amyloid protein binds to the affinity column is likely to be due to its inherent affinity for the agarose-based matrix, since it binds efficiently to sugar-based columns in a Ca^2+^-dependent manner (de Beer and Pepys 1982). Each polypeptide in the serum amyloid protein pentamer bears a single N-linked glycan, but these glycans are essentially fully sialylated (Pepys et al. 1995) and thus would not be good ligands for BDCA-2.

### Characterization of immunoglobulin binding to BDCA-2

**Fig. 2.**
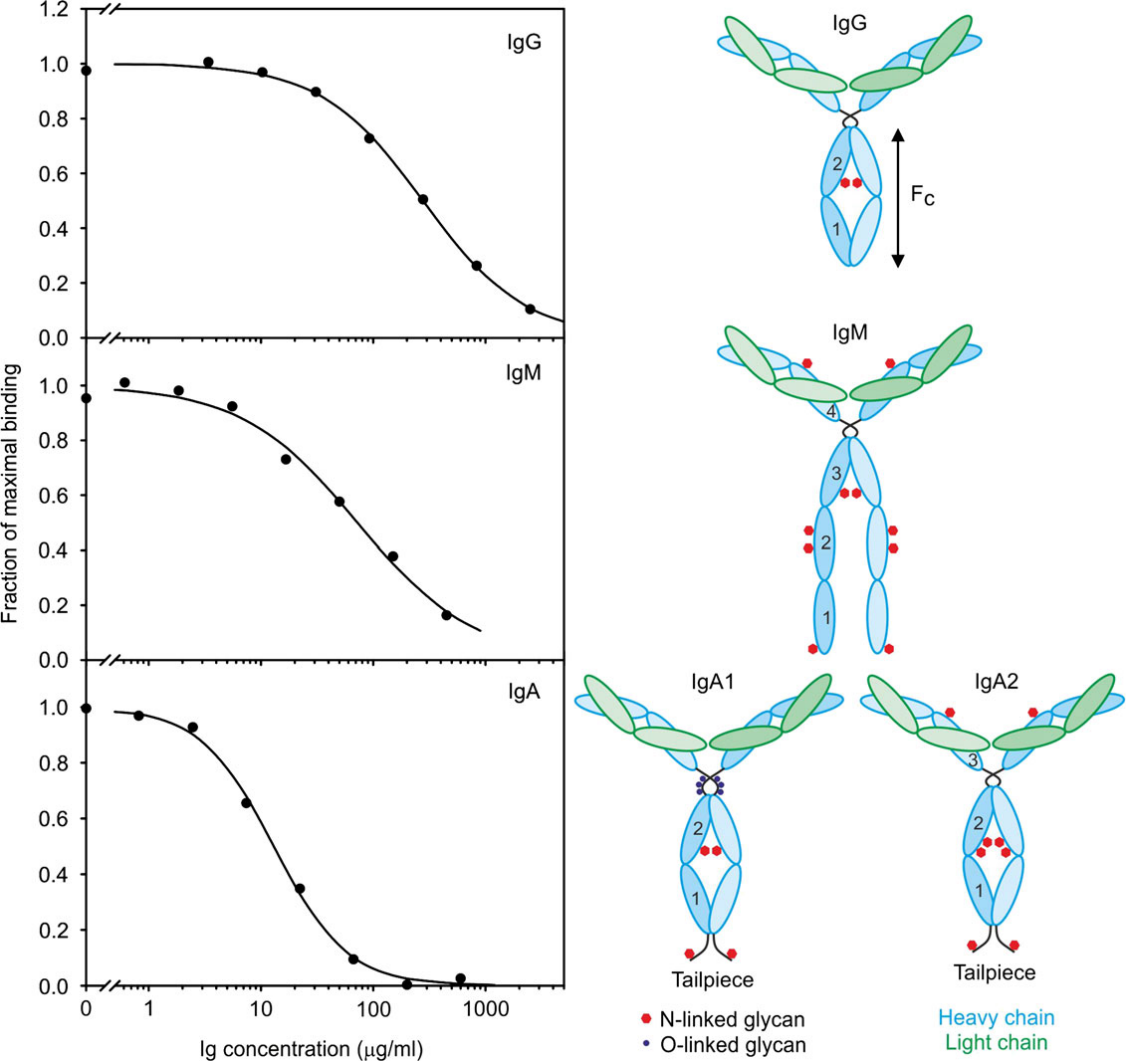
Binding competition assays for different classes of immunoglobulin binding to BDCA-2. Biotin-tagged CRD from BDCA-2 was immobilized on streptavidin-coated plates that were probed with ^125^I-Man-BSA.in the presence of different concentrations of competing glycoprotein ligands. Experimental values, which are the average of duplicates, are shown as circles. *K*_I_ values were determined by nonlinear least squares fitting to a simple binding competition equation, with the fitted curve shown as a continuous line in each graph. Example assays are shown. IgA used in these assays was purified from human serum by affinity chromatography on anti-IgA antibody. The locations of glycosylation sites in each of the three classes of immunoglobulins are shown at the right.

Affinities of the serum immunoglobulins for BDCA-2 were compared in binding competition assays (Figure 2). Biotin-tagged CRD immobilized in 96-well plates coated with streptavidin was probed with the neoglycoprotein reporter ligand, ^125^I-labeled Man_23_-BSA (bovine serum albumin). Although Galβ1-4GlcNAcα1-2Man is an optimal glycan ligand for BDCA-2, the mannose residue occupies the primary binding site in the CRD and can bind as a monosaccharide with lower affinity. Thus, the reporter ligand can be used at subsaturating concentrations, so the radioactivity detected is proportional to the amount of CRD not complexed with competing glycoprotein ligand. Inhibition constants (*K*_I_) obtained under these conditions are close approximations to dissociation constants (*K*_D_) for the competing ligands (Levitzki 1997).

The results reveal significant differences in the affinities of the different immunoglobulins. IgA is the highest affinity glycoprotein ligand and IgG is the weakest. A comparison of the *K*_I_ values for the different immunoglobulins in serum with their concentrations in serum reveals that while IgG is the most abundant glycoprotein ligand, it also has the lowest affinity (Table II). The comparison also reveals that the serum concentrations are significantly higher than the *K*_D_ values for each of the immunoglobulins, suggesting that the receptor on circulating plasmacytoid dendritic cells would be largely saturated with immunoglobulins.

**Table II.** Affinities of immunoglobulins for BDCA-2 and concentrations in serum

Glycoprotein ligand	*K*_I_ value (μg/mL)	Serum concentration (mg/mL)	Ratio
IgG	263 ± 45	10.5	40
IgM	96 ± 18	1.4	15
IgA	12 ± 1	2.3	190

*K*_I_ values are summarized as averages from three or more binding competition experiments, each performed in duplicate. The ratio is the serum concentration (Gonzalez-Quintela et al. 2007) divided by the *K*_I_ value.

**Fig. 3.**
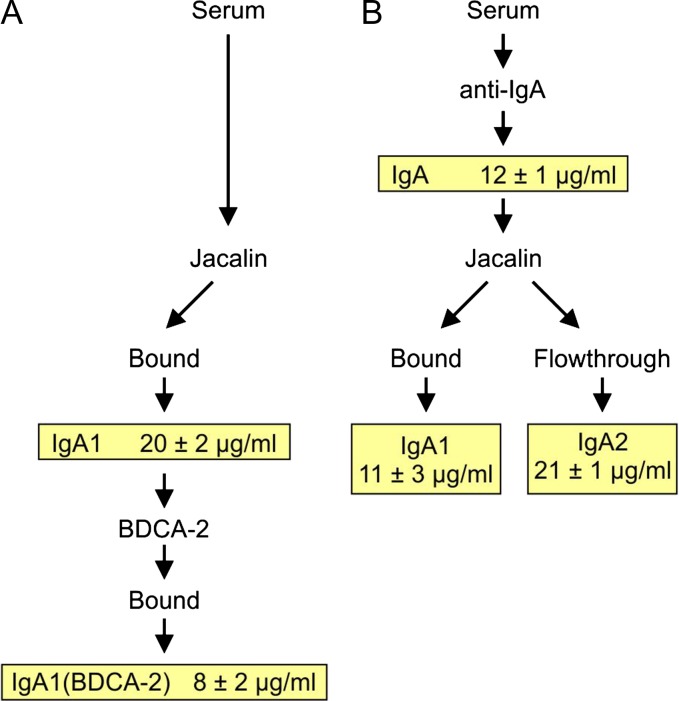
Summary of BDCA-2 binding to IgA1 and IgA2 prepared by different methods. (**A**) Direct purification of IgA1 from serum using the plant lectin jacalin, which binds to the O-linked glycans present in the hinge region of IgA1 but not IgA2 (Figure 2). The IgA1 pool was eluted at low pH and further fractionated by chromatography on the CRD from BDCA-2 immobilized on agarose resin. IgA1 that bound to BDCA-2 was eluted with EDTA. (**B**) Purification of total IgA from serum by chromatography on immobilized anti-IgA antibodies, followed by fractionation of IgA1 and IgA2 by chromatography on jacalin. The purification schemes are shown schematically, along with the *K*_I_ values, determined using the binding competition assay described in Figure 2, for the different preparations. *K*_I_ values represent the mean ± standard deviation for three experiments, each performed in duplicate.

Because IgA exists in two forms that differ significantly in glycosylation, it was of interest to compare the affinities of the IgA1 and IgA2 isotypes (Woof and Russell 2011). Several schemes were employed to isolate the two forms, either directly from serum or by fractionation of the total IgA pool (Figure 3). The affinities for BDCA-2 were then measured separately in the binding competition assay. Because IgA1 predominates in serum (Delacroix et al. 1982), the affinity of the total IgA pool reflects largely the affinity of IgA1. The affinity of IgA2 is approximately 2-fold lower than the affinity for IgA1 in spite of the presence of additional N-linked sites in IgA2 compared to IgA1. As noted above, differences in affinities of the different classes of immunoglobulins represent a combination of the effects of different amounts of appropriate galactose-terminated glycan ligands present on the different heavy chains and the accessibility of these glycans. In both forms of IgA, an N-linked glycan attached to an N-terminal tail piece on the heavy chain (Figure 2) is likely to be exposed and may form the dominant binding site for BDCA-2. The absence of a similar site in the IgG heavy chain is consistent with the lower affinity of BDCA-2 for IgG compared to IgA.

The possibility that binding to IgA might reflect the presence of a small proportion of the protein that bears an unusual glycan with very high affinity was examined by fractionation of the IgA on the immobilized BDCA-2 affinity column. Binding competition experiments with the material eluted from BDCA-2 with EDTA showed that the affinity of this subfraction was only modestly enhanced compared to the total pool of IgA (Figure 3A). Thus, there does not appear to be a small pool of very high-affinity forms. Instead, a significant fraction of the IgA molecules bear glycans that are ligands for BDCA-2.

### Mechanism of glycoprotein ligand binding to BDCA-2

**Fig. 4.**
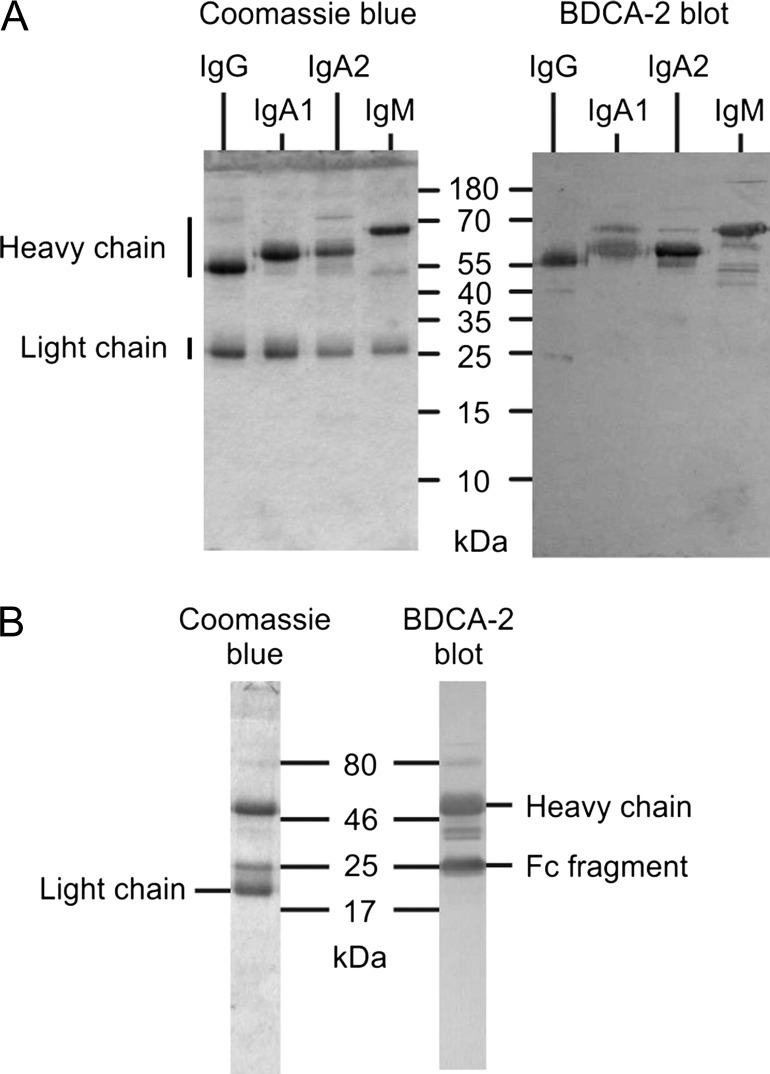
Binding of BDCA-2 to glycans on heavy chains of immunoglobulins. (**A**) Approximately 2 μg of each immunoglobulin was separated on 17.5% SDS polyacrylamide gels, which were either stained with Coomassie blue or blotted onto nitrocellulose and probed with BDCA-2–streptavidin–alkaline phosphatase. IgA1 and IgA2 were resolved as shown in Figure 3B. (**B**) Binding of BDCA-2–strepatividin–alkaline phosphatase complex to fragments of IgG following papain digestion. Following partial digestion of IgG with papain, the remaining fragments were purified on immobilized protein A and separated on 17.5% SDS polyacrylamide gels. Parallel lanes were stained with Coomassie blue and blotted onto nitrocellulose for probing with BDCA-2–strepatividin–alkaline phosphatase complex.

The presence of glycan ligands on the heavy chains of IgG, IgM and IgA was confirmed by probing a blot of the immunoglobulin preparations with BDCA-2 (Figure 4A). The results demonstrate the presence of glycan ligands on each of the heavy chain polypeptides. Based on the previous glycan array results, the main oligosaccharide ligands for BDCA-2 would be expected to be bi-antennary N-linked structures bearing terminal galactose residues (Jégouzo et al. 2015). In the case of IgG, the predominant glycans attached to the second constant domain in the Fc region of the polypeptide have been extensively characterized. More than 25% of these glycans have terminal galactose residues on each branch (Yamada et al. 1997). In order to confirm that glycans in the Fc region are the ones that interact with BDCA-2, IgG was subjected to papain digestion to release the Fc fragment and binding to the resulting fragment was detected following SDS polyacrylamide gel electrophoresis, blotting and probing with BDCA-2–streptavidin–alkaline phosphatase complex (Figure 4B). The results confirm that the binding is through the glycan attached to the Fc region.

**Fig. 5.**
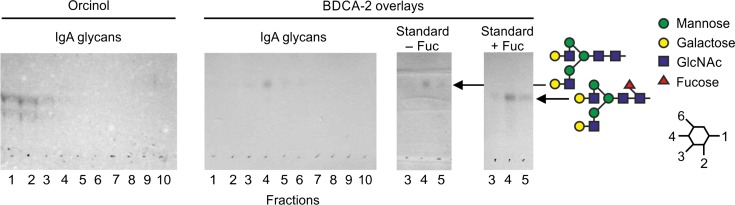
Analysis of neoglycolipids derived from IgA glycans. Glycans released from IgA by digestion with protein N-glycosidase were attached to a synthetic analog of phosphatidyl ethanolamine by reductive amination The resulting neoglycolipids were fractionated by reverse phase chromatography in CHCl_3_/methanol/water 60:35:8. Aliquots of the resulting fractions were separated on high-performance silica thin-layer chromatography plates in CHCl_3_/methanol/water 105:100:28. Left, thin-layer chromatogram stained with orcinol showing the distribution of total neoglycolipids for the full set of elution fractions. Right, chromatograms were soaked with polyisobutylmethacrylate and probed with BDCA-2–strepatividin–alkaline phosphatase complex. Galactose-terminated bi-antennary glycans were processed in parallel for comparison. These standards elute in fraction 3–5.

Because glycosylation of IgA is more complex than IgG, with multiple sites both within the Fc region and in the N-terminal extension, the types of glycans involved in binding were analyzed directly. Glycans released from purified IgA by digestion with protein N-glycosidase were coupled to a synthetic lipid anchor. Following resolution by thin-layer chromatography, the neoglycolipids were probed with BDCA-2–streptavidin–alkaline phosphatase complex (Figure 5). The predominant glycan that interacts with BDCA-2 co-migrates with the standard galactose-terminated bi-antennary glycan. A second slower migrating species has the mobility of the fucosylated form of this glycan. The neoglycolipids that bind to BDCA-2 represent a minor component of the total glycan pool which is barely visible following orcinol staining for all sugars. Taken together, the results suggest that selective binding of immunoglobulins reflects the presence of relatively high amounts of bi-antennary, galactose-terminated glycans rather than a previously unidentified glycan with higher affinity.

### Characterization of α_2_-Macroglobulin binding to BDCA-2

**Fig. 6.**
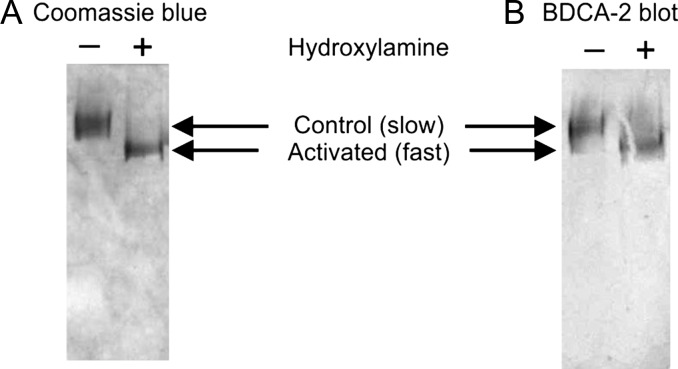
α_2_-Macroglobulin native and activated gel blot. Purified α_2_-macroglobulin was separated by native polyacrylamide gel electrophoresis before and after treatment with hydroxylamine. The hydroxylamine treatment results in conversion to the electrophoretically fast form. (**A**) Coomassie blue stained gel. (**B**) Gel blotted onto nitrocellulose and probed with BDCA-2–streptavidin–alkaline phosphatase complex.

α_2_-Macroglobulin bears a complex range of glycans (Arnold et al. 2006). Nevertheless, neoglycolipid analysis reveals that, as in the case of the immunoglobulins, the oligosaccharides that bind to BDCA-2 are predominantly the fucosylated and nonfucosylated forms of the galactose-terminated bi-antennary glycan (data not shown). In serum, α_2_-macroglobulin circulates in an unactivated state, with an exposed cavity primed for interaction with proteases. When a protease enters the cavity and cleaves the bait region, the cleavage triggers a conformational change that entraps the protease. After the conformational change, the α_2_-macroglobulin is subject to accelerated clearance from circulation (Borth 1992). It has been suggested that the conformational change might change the accessibility of glycans on the surface of the α_2_-macroglobulin (Imber and Pizzo 1981). Thus, it was of interest to determine if activation of α_2_-macroglobulin results in a change in its interaction with BDCA-2. However, blotting with BDCA-2 produced similar results for α_2_-macroglobulin as purified from serum and following activation by treatment with methylamine (Figure 6). In the binding competition assay, the *K*_I_ values for the untreated and treated forms were 43 ± 4 and 48 ± 18 μg/mL. It therefore seems unlikely that BDCA-2 responds to α_2_-macroglobulin activation. Nevertheless, as in the case of the serum immunoglobulins, the concentration of α_2_-macroglobulin in serum of ~3 mg/mL (Housley 1968) is significantly higher than the measured binding affinity for BDCA-2.

## Discussion

The discovery of serum glycoprotein ligands for BDCA-2 raises several questions about the mechanistic basis for the observed affinities and the biological significance of the binding interactions. Comparing the results of different assays for binding provides insights into the significance of both the abundance of the specific glycans with highest affinity for the CRD in BDCA-2 and their degree of exposure on native glycoproteins. The neoglycolipid experiments indicate that the dominant glycan ligands are bi-antennary, galactose-terminated oligosaccharides. While the mono-galactosylated form can bind (Jégouzo et al. 2015) and is present on IgG and IgA in abundance similar to that of the bi-galactosylated form (Yamada et al. 1997), binding only to the bi-galactosylated form is observed (Figure 5).

An interesting question with all of the immunoglobulins is the fact that terminal galactose residues are exposed, allowing binding to BDCA-2, yet the glycoproteins are not cleared from circulation by the hepatic asialoglycoprotein receptor. The major factor in this differential recognition is likely to be the preponderance of bi-antennary glycans on the immunoglobulins, since more branched glycans lead to substantially more efficient clearance by the hepatic receptor (Rice and Lee 1993). Thus, the glycan composition can be tuned to facilitate interaction with one receptor but not another.

When the glycoproteins are displayed in denatured form on nitrocellulose, the most prominent glycoprotein ligand is IgG (Figure 1A). This binding reflects the abundance of IgG in serum and the presence of the bi-galactosylated glycan ligand, which represents in excess of 25% of the glycans attached to the Fc domain (Yamada et al. 1997). In contrast, following purification on the BDCA-2 affinity column, the IgG is barely detected and IgA is significantly enriched, although only about 10% of the glycans attached to IgA1 are likely targets for BDCA-2 (Oortwijn et al. 2006). The quantitative binding experiments also indicate that IgG binds with more than 20-fold lower affinity than IgA. It is likely that these differences reflect the different placement of the N-linked glycans on IgG and IgA. The single glycan on an IgG heavy chain is attached to the second constant domain of the Fc fraction and is largely sequestered in a cavity within the protein. Thus, these glycans would be hidden for a significant fraction of the time on the native protein, although they would be available for efficient binding following SDS polyacrylamide gel electrophoresis and blotting. In contrast, one of the several N-linked glycans attached to the heavy chain of IgA is appended to an asparagine residue in a 17-amino acid C-terminal tail piece. Scattering data and molecular dynamics calculations indicate that in the predominant, monomeric serum form of IgA this portion of the polypeptide is exposed and flexible (Hui et al. 2015), so the attached glycan would be accessible for BDCA-2 binding even on the native protein. Unfortunately, the exact structures of the glycans attached at this site are not known.

The affinity for IgM lies between that of IgA and IgG. Compared to the abundance of the proteins, the signal for binding of BDCA-2 to IgM heavy chain in the gel blotting format is relatively weaker than that of the less abundant IgA. On the BDCA-2 affinity column, IgM is enriched but again not as much as IgA. There is an N-linked glycan attached to a C-terminal tail piece in the heavy chain of IgM as in IgA, but in circulating IgM the tail piece is largely complexed with J chain and is thus less exposed. Although glycosylation at this site is not fully characterized, it appears to be incomplete and poorly processed and the bi-antennary complex glycan that is a target for BDCA-2 has not been detected at this site (Hong et al. 2015; Moh et al. 2016).

The various in vitro experiments described here define structural features of glycoproteins that make them ligands for BDCA-2, but additional factors, such as appropriate geometry, density and valency may be needed in order to stimulate BDCA-2 activity. If the serum glycoprotein ligands can initiate signaling through the FcRγ subunit, then under normal conditions in vivo, the baseline level of activation would be high. Even under typical culture conditions for plasmacytoid dendritic cells, 10% serum could induce substantial stimulation. Assays for stimulation with monoclonal antibodies conducted in the absence of serum would be starting from an artificially low level of receptor occupancy. One possibility is that the serum glycoprotein ligands maintain the plasmacytoid dendritic cells in a quiescent state and that the inhibition is relieved when the cells leave the circulation. Interestingly, plasmacytoid dendritic cells in the skin lesions of patients with systemic lupus erythematosus are an important source of type I IFN (Pellerin et al. 2015). IFN secretion can be reduced by an antibody to BDCA-2. However, in addition to inhibiting IFN secretion, the antibody causes internalization of BDCA-2, so it is unclear to what extent natural serum glycoprotein ligands would mimic this activity of the antibody. Alternatively, if the serum glycoprotein ligands do not stimulate signaling, they would inhibit stimulation by competing with other ligands.

The finding that BDCA-2 binds to IgG and other immunoglobulins may provide a potential link between level of glycosylation of serum glycoproteins and activity of the immune system. It is well established that there is a decreased galactosylation of IgG in various diseases, particularly in inflammatory conditions like arthritis (Huhn et al. 2009). Decreased galactosylation would decrease the level of binding of serum glycoprotein ligands to BDCA-2, potentially relieving the inhibitory signaling in the plasmacytoid dendritic cells. Conversely, there is an increase in galactosylation during pregnancy, correlating with increased immune tolerance. Increased binding of galactosylated IgG to BDCA-2 would suppress activation of the dendritic cells. The results reported here suggest the possibility that the ability of BDCA-2 to sense serum glycosylation may play an important role in this type of immunomodulation. Thus, it will eventually be of interest to examine the ability of the immunoglobulins to bind to BDCA-2 on the surface of cells and to initiate intracellular signaling.

## Materials and methods

### BDCA-2 production and immobilization

Both biotin-tagged and untagged CRD from BDCA-2 were expressed in *Escherichia coli* and purified over an egg-glycopeptide column as previously described (Jégouzo et al. 2015). A CRD–streptavidin–alkaline phosphatase complex was created by incubating 200 μg of streptavidin–alkaline phosphatase (Sigma Chemical Company, Dorset, UK) with 500 μg of biotin-tagged CRD overnight at 4°C. Ca^2+^ was added to a concentration of 25 mM and the complex was repurified on a 2-mL mannose-Sepharose column (Jégouzo et al. 2015) which was rinsed with 6 mL of loading buffer (150 mM NaCl, 25 mM Tris-Cl, pH 7.8, 25 mM CaCl_2_) and eluted with 1-mL aliquots of elution buffer (150 mM NaCl, 25 mM Tris-Cl, pH 7.8, 2.5 mM EDTA). The uncomplexed CRD binds poorly to the mannose-Sepharose and rinses through the column in the presence of Ca^2+^ (Jégouzo et al. 2015), while the tetrameric complex remains bound and is eluted with EDTA.

An affinity column was created with the untagged CRD. Purified CRD was dialyzed extensively against coupling buffer: 150 mM NaCl, 10 mM HEPES, pH 8.0, 25 mM CaCl_2_. A solution of 2.5 mg of the protein in 10 mL of coupling buffer with 1.2 mL of Affigel 10 resin (BioRad Laboratories, Watford, UK) was incubated for 4 h at 4°C with end-over-end mixing. Unbound protein was drained from the column. Based on SDS polyacrylamide gel analysis of the coupling solution before and after incubation with the resin, the coupling efficiency was nearly 100%. The coupled resin was incubated with high-salt loading buffer (0.5 M NaCl, 25 mM Tris-Cl, pH 7.8, 25 mM CaCl_2_) overnight at 4°C to block any remaining N-hydroxysuccinimide groups.

### Isolation of BDCA-2 glycoprotein ligands from serum

An aliquot of human serum (250 μL; Sigma) was mixed with an equal volume of high-salt loading buffer, spun for 5 min at 18,000 × *g* and loaded onto the BDCA-2 affinity column equilibrated in high-salt loading buffer. The column was washed with 6 mL of high-salt loading buffer followed by 6 mL of loading buffer and was eluted with eight 0.5-mL aliquots of elution buffer.

### Proteomic analysis

For proteomic analysis, eluted fractions were pooled and sufficient 30% trichloroacetic acid was added to achieve a final concentration of 10%. After incubation on ice for 10 min, protein was collected by centrifugation for 5 min at 18,000 × *g*. The pellet was washed twice with 250 μL of 50:50 ethanol/ether, dried under vacuum and dissolved in SDS gel sample buffer by heating at 100°C for 5 min. Proteins were resolved on a 12.5% SDS polyacrylamide gel and stained with Coomassie blue. For proteomic analysis, bands were excised and subjected to reduction and carboxymethylation and trypsin digestion (Powlesland et al. 2009). MS data as well as MS/MS data for the 10 most abundant ions in each sample were collected on an Applied Biosystems 4800 matrix-assisted laser desorption ionization time-of-flight mass spectrometer using GPS Explorer software version 3.6 (Applied Biosystems, Foster City, CA). Version 2.2 of the Mascot database search algorithm (www.matrixscience.com) was used to search the human subset of the National Center for Biotechnology Information protein database using the parameters described previously (Powlesland et al. 2009). Probability-based Mowse protein scores >42 were considered significant (*P* > 0.05) (Perkins et al. 1999).

### IgG and IgA fractionation

IgG at a concentration of 5 mg/mL was treated with 75 μg/mL papain (Sigma) in 100 mM sodium acetate, pH 5.2, 1 mM EDTA, 50 mM cysteine for 60 min at 37°C. The reaction was stopped by the addition of iodoacetic acid to a final concentration of 75 mM, which was allowed to react for 15 min at room temperature. The remaining intact protein and Fc fragment were bound to a 1-mL column of immobilized protein A (GE Life Sciences, Little Chalfont, UK) which was washed with 10 volumes of 100 mM Tris-Cl, pH 7.5 and eluted with 100 mM glycine, pH 3.0.

Total IgA purified from human serum by anti-IgA affinity chromatography was purchased from Sigma. Alternatively, total IgA was purified from human serum (Sigma) on immobilized staphylococcal superantigen-like protein 7 (Invivogen, Toulouse, France) following the manufacturer’s instructions (Langley et al. 2005). Affinity chromatography on Jacalin agarose (Pierce Chemical Company, Hemel Hempstead, UK) was used to purify IgA1 directly from serum and to fractionate total IgA pools into IgA1 and IgA2 (Gregory et al. 1987; Pack 2000).

### α_2_-Macroglobulin purification and activation

α_2_-Macroglobulin was isolated from human serum based on a published protocol (Arnold et al. 2006) with minor variations. Human serum (10 mL) was dialyzed overnight against water and centrifuged at 100,000 × *g* for 30 min before application to a 2-mL column of nitrilotriacetic acid–agarose resin charged with Zn^2+^. The column was rinsed with sodium phosphate buffers at pH 7.0 and 6.0 and eluted with at pH 5.0. Fractions containing α_2_-macroglobulin identified by SDS polyacrylamide gel electrophoresis on 10% polyacrylamide gels were pooled and further fractionated on a 1 × 30 cm Superdex S200 column eluted with 150 mM NaCl, 10 mM Tris-Cl, pH 7.8 at a flow rate of 0.5 mL/min. Fractions containing pure α_2_-macroglobulin identified by SDS polyacrylamide gel electrophoresis were pooled. Aliquots were activated by incubation with 0.2 M methylamine for 4 h at room temperature, followed by repurification by gel filtration on the Superdex S200 column. Unactivated α_2_-macroglobulin was also rerun on the column in parallel. Activation was confirmed by native polyacrylamide gel electrophoresis.

### Gels and blotting

SDS polyacrylamide gel electrophoresis was performed by the method of Laemmli (Laemmli 1970) followed by staining with Coomassie blue or blotting onto nitrocellulose. Samples of fetuin and asialofetuin were obtained from Sigma. Blots were blocked for 30 min at room temperature with 5% BSA in binding buffer: 150 mM NaCl, 25 mM Tris-Cl, pH 7.8, 2.5 mM CaCl_2_. CRD–streptavidin–alkaline phosphatase complex was added to a final concentration of 5–10 μg/mL and incubated for 60 min at room temperature. Following four 5-min washes with binding buffer, blots were developed with nitroblue tetrazolium/5-bromo-4-chloro-3-indolyl phosphate (Merck, Watford, UK). Native gel electrophoresis was performed on 5% polyacrylamide gels using the Laemmli buffer system but without SDS.

### Neoglycolipids

Neoglycolipids were generated by the reduction of the Schiff base formed between the reducing-end sugar of oligosaccharides and the dipalmitoyl phosphatidyl ethanolamine analog 1,2‐dihexadecyl‐*sn*‐glycero‐3‐phosphoethanolamine (Sigma) (Liu et al. 2006). Neoglycolipids were separated from excess lipid by chromatography on 225 mg Oasis HLB plus reverse phase cartridges (Waters, Elstree, UK) prewashed with 2 mL of methanol:water 1/1. The dried reaction was dissolved in 100 μL of CHCl_3_:methanol:water 15/70/30 by heating briefly to 60°C and applied to the cartridge, which was rinsed with 2 × 1 mL of water and 1 mL of methanol before elution with 200-μL aliquots of CHCl_3_/methanol/water 60:35:8. Aliquots (5 μL) of each fraction were resolved on aluminum-backed high-performance silica gel chromatography plates (Merck) developed with CHCl_3_/methanol/water 105:100:28, which were either stained with orcinol or fixed in place by soaking the plate for 30 s in a 0.1% solution of polyisobutylmethacrylate in hexane and air drying. The fixed plate was blocked with 5% BSA in binding buffer and visualization with CRD was performed as for the gel blots. Reference bi-antennary oligosaccharides were obtained from Dextra Laboratories.

### Competition binding assays

For solid-phase competition binding assays, the biotin-tagged CRD of BDCA-2 was bound to streptavidin-coated wells (Pierce) and probed with ^125^I-labeled Man_33_-BSA as previously described (Jégouzo et al. 2015). Each assay was performed in duplicate and final *K*_I_ values represent averages ± standard deviations for at least three assays.

## Abbreviations

BDCA-2: blood dendritic cell antigen 2
IFN: interferon
CRD: carbohydrate recognition domain
FcRγ: Fc receptor γ subunit
EDTA: ethylenediaminetetraacetic acid
BSA: bovine serum albumin
